# Gender Differences in the Influence of Social Support on One-Year Changes in Functional Status in Older Patients with Heart Failure

**DOI:** 10.1155/2012/616372

**Published:** 2012-08-22

**Authors:** Danielle M. Berard, Elizabeth G. VanDenKerkhof, Margaret Harrison, Joan E. Tranmer

**Affiliations:** School of Nursing, Queen's University, Kingston, ON, Canada K7L 3N6

## Abstract

The purpose of this study was to determine the combined effects of gender and levels of social support on 1-year functional health outcomes in older persons diagnosed with heart failure (HF). Persons ≥ 65 years of age with an acute HF exacerbation (164 females; 271 males) were enrolled and followed for a year. Participants completed baseline and 12-month questionnaires containing clinical and demographic descriptive information and validated self-report measures of: (1) physical functioning (Medical Outcome Study [MOS] SF12 and Kansas City Cardiomyopathy Questionnaire [KCCQ]) and (2) social support (MOS- Social Support Survey). Women were more likely to be single, widowed or divorced, living alone and earning less annual income. At baseline, women reported significantly lower support and physical function scores. However, at 1 year there were no significant gender differences in the proportion of men or women who experienced clinically meaningful functional decline or death across the year of follow-up. In multivariable modeling, men with lower levels of social support were more likely to experience functional decline. This was not the case for women. Our findings suggest that gender-directed strategies to promote optimization of function for both men and women living with HF in their community are warranted.

## 1. Introduction

Heart failure (HF) is a common chronic cardiovascular disease that typically presents as episodes of acute exacerbation combined with periods of clinical stability. HF affects all ages, but in particular, is a disease of older adults. Due to its chronic nature, patients and their caregivers assume much of the daily management; thus, it is important to understand the influence of nonmedically related care factors, such as social support, on health outcomes and functionality. We know that the personal, clinical, and social profiles of persons with chronic conditions such as heart failure will vary. Older women are more likely to (a) have limited social supports, (b) be living on their own, with less financial resources, (c) not access formalized supports such as cardiac rehabilitation programs, (d) report poorer health-related quality of life, and (e) have worse physical function, in comparison to men [1–3]. Research evidence also suggests that poor levels of social support are associated with mortality and other adverse outcomes in persons with cardiac disease [4–8]. Social support is often contextualized as interpersonal transactions that provide functional support consisting of (a) emotional support (involving care, love, and empathy), (b) instrumental or tangible support (goods and services), (c) informational support (including guidance or feedback or environmental information), or (d) appraisal (information specifically related to self-evaluation and care) [2]. Some postulate that social support facilitates coping and adaption and moderates the psychological and physiological consequences of illness [9, 10].

Given that high levels of social support may promote psychological and physical well-being and good health behaviours, it is unclear whether gender differences and varying levels of social support or a combination of these factors influence functional well-being and other health outcomes for older persons with HF [11]. Therefore, the purpose of this study was to describe the effects of gender differences and social support on health outcomes, while controlling for personal demographics, disease severity, and comorbid conditions. 

## 2. Methods

We conducted a prospective cohort study with one-year followup. The Queen's University Health Sciences and Affiliated Teaching Hospitals Research Ethics Board (Kingston, Ontario) reviewed and approved the study protocol. 

### 2.1. Participant Sample

Potential participants were recruited from one tertiary teaching centre in Kingston, Ontario, and 3 community hospital corporations in the surrounding region. Recruitment was carried out between March 2003 and September 2007; followup continued until January 2009. Participants were included if they were aged 65 years and older and seen in the emergency department (ED) with a diagnosis of HF or HF-related complaint. The HF diagnosis was confirmed through chart review in accordance with the Framingham Criteria for Congestive Heart Failure [12]. Participants were excluded if they lived in such institutions as nursing homes, or long-term care facilities. 

The study cohort consisted of 435 study participants who provided informed consent and completed baseline questionnaires. Given the observed gender distribution and loss to follow-up rate, our effective sample size achieves over 85% at a two-sided alpha = .05 to compare functional decline rates between genders if the true absolute difference is at least 10%. Furthermore, our effective sample size provides at least 80% power at alpha = .05 (two-sided) to test the association between social support and functional decline if a one standard deviation change in the social support subscale results in at least 10% difference in the proportion of patients who decline (regardless of outcome). 

### 2.2. Data Collection Procedure

All consecutive ED discharge records were reviewed for potential participants who met inclusion criteria. Once informed consent was obtained, baseline information was collected either in hospital or obtained after discharge. Participants completed self-report questionnaires at baseline and 12 months in home and returned the package in a self-addressed envelope. If questionnaires were not returned at the designated time interval, the research coordinator contacted the participant and encouraged the participants to complete the questionnaire. In some cases, the participant provided questionnaire responses over the phone and the research coordinator completed the questionnaire. Data were entered into a secure computerized data base system maintained in the Nursing Research Unit at Kingston General Hospital, Kingston, Ontario. Data entry accuracy was verified by a second research associate. During the one-year follow-up period, survival status was determined through hospital records or through phone contact with family. 

## 3. Measures

### 3.1. Primary Outcome: Physical Function

The primary outcome of interest was clinically important changes in physical function as related to (a) heart disease, measured by the physical limitation (PL) subscale score from the Kansas City Cardiomyopathy Questionnaire (KCCQ), or (b) overall health-related quality of life as measured by the physical component summary scale (PCS) of the SF-12 Health Survey. 

The *Kansas City Cardiomyopathy Questionnaire (KCCQ)* is a disease-specific, 23-item questionnaire that quantifies the following domains: physical limitation, symptoms (frequency, severity, and recent change), social limitation, self-efficacy and knowledge, and quality of life. Scale scores are transformed to a 0 to 100 range by subtracting the lowest possible scale score, dividing by the range of the scale and multiplying by 100. This tool is a valid, reliable self-reported health status measure for patients with HF. Cronbach's alphas for each domain indicate high internal consistency, except for self-efficacy that has moderate internal consistency (.62) [13]. As we were most interested in physical function, we focused solely on the physical limitation (PL) score of the KCCQ. Based on the literature, we defined clinically important changes in PL as a 5-point change in score in either direction between baseline and 12 months [14, 15]. Participants were then classified accordingly: (1) improvement or maintenance of physical function, (2) decline in physical function, (3) death within the 12-month followup, (4) withdrawal from the study due to worsening illness, and (5) lost to followup/withdrawal for unknown reasons [14–16]. 

The physical component summary scale (PCS) of the SF-12 Health Survey was selected as a brief, patient-reported outcome for overall physical health status. The SF-12 is reliable and has been a validated tool used to measure self-reported generic functioning and well-being in numerous medical and surgical populations [17, 18]. Mean reliability coefficients are reported between .64 and .87 for the physical dimension. Using PCS scores at baseline and 12 months, a similar 5-level categorical outcome variable was created based on clinically significant changes. Based on the literature, we used a 2-point change in PCS to reflect a clinically significant change in function [19, 20]. Similar to the KCCQ-PL categorical classification, participants were classified into the 5 levels using the PCS change score. 

### 3.2. Exposure Variable of Interest: Social Support

Social support was measured using the Medical Outcome Survey-Social Support Survey (MOS-SSS). The MOS-SSS is a 20-item self-report tool that measures four aspects of functional support including emotional/informational, tangible, affectionate, and positive social interaction [21]. Scores range from 0 to 100, with higher scores indicating higher levels of perceived support. Bennett and colleagues [2] used the MOS-SSS to measure social support to determine associations between support and health-related quality of life in 227 hospitalized HF patients. Mean overall scores (±SD) at baseline were 56 (±18.2) and 53 (±20.1) at 12 months, with a score of 76 indicating positive perceptions of support [2]. 

### 3.3. Personal Characteristics

Age, gender, income, educational level, and health habits were recorded at baseline by self-report and were used to describe the sample. Home supports such as living arrangements, housing arrangements, and marital status were also collected, as well as health supports including access to a family physician, cardiologist/internist, details about who manages the HF, and use of HF resources.

### 3.4. Clinical Characteristics

Left ventricular ejection fraction (LVEF) of <40% was our primary measure of disease severity. This value was obtained from echocardiogram history during a chart review. As well, we determined to what extent participants met the Framingham Criteria for HF (major or minor). Other diseases that potentially influenced participants' functionality were identified using the Functional Comorbidity Index (FCI), a validated, self-reported tool used to identify 17 common comorbid conditions used to predict one's level of physical functional capacity [22, 23]. This tool has been validated on a cross-sectional database of 9,423 Canadian adults, using the SF-36 physical function subscale as the outcome measure [24].

## 4. Analysis

Data were analyzed using SPSS Version 17.0 software. All baseline and 12-month covariate and social support scales were described using standard descriptive statistics (means, standard deviation; frequencies and percentages). When 15% of any questionnaire data were not obtained, the survey data for that particular participant were considered missing. We determined the gender differences in personal and clinical variables and social support using the Chi-squared test for categorical variables and the *t*-test for normally distributed continuous variable scores. Logistic regression models were estimated for each subscale and overall social support score using a two-level functional outcome for each of the PL and PCS change scores: (1) maintained functional status or improved by the minimal clinically important difference and (2) died, lost to followup due to worsening illness or declined by the minimal clinically important difference. These models controlled for age and gender and were determined in the full sample and within each gender. The effect modification of gender on social support was determined through inclusion of an interaction term in each model.

The large number of participants lost to followup was addressed in two ways. First, we compared the baseline characteristics of those participants with complete 12-month data to those participants with incomplete data at 12 months, to determine if the groups were different. Secondly, we conducted a sensitivity analyses in which we compared the logistic regression model in 2 cohorts: (1) a cohort that did not include those participants that were lost to followup and (2) a cohort that included the loss to followup in the declined/died/too ill category. It should be noted that for some individuals we were able to categorize participants within the functional decline category, without questionnaire data, as they reported to the study coordinator their intent to withdraw due to worsening illness and decline. This sensitivity analysis allowed us to test the assumption that those participants lost to followup could be included in the declined/died/too ill adverse outcome group. 

## 5. Results

Four hundred and thirty-five participants provided baseline information; 224 (52%) completed all or parts of the questionnaires at 12 months. Of the 435 who started, many eligible and consenting participants did not complete the study due to worsened illness (*n* = 30), death (*n* = 54), admission to assisted living facilities (*n* = 4), or other unknown reasons (*n* = 123). There were few significant differences in baseline characteristics between those who completed the study and those who were lost to followup (Table 1). Participants who completed the study were more likely to report higher KCCQ physical limitation scores than those who did not complete the follow-up questionnaires. Because of these results, we considered the lost-to-followup group as potentially different from those who completed, and we therefore included a separate classification for this group in relevant analyses. 

Baseline participant characteristics of the total sample (completers and noncompleters combined) are described in Tables 2(a) and 2(b). The study cohort included 62% males aged 65–99 years. Female participants were more likely to be single, widowed or divorced, living alone, and earning less annual income (*P* < .01). At baseline, men were more likely to have a LVEF ≤ 40% (*P* < .01). There were no sex differences in both the major and minor criteria for heart failure (Table 2(b)). The number of comorbid conditions were not significantly different between genders but were ranked differently with women reporting higher prevalence of asthma, angina, visual impairment, and depression and men reporting degenerative disc disease, myocardial infarction, diabetes, lung disease, and hearing impairment.

### 5.1. Gender Differences in Social Support

Table 3 presents the mean subscale and overall social support scores at baseline. Mean (SD) scores for females ranged from 67.3 (31.7) to 80.9 (27.0), with affectionate support being the highest subscale score. Mean (SD) scores for males ranged from 72.3 (32.9) to 80.4 (30.4), with tangible and affectionate support being the highest subscale scores. Women tended to report lower scores overall, but only tangible support was significantly lower (*P* < .01).

### 5.2. Functional Outcomes

Table 4 presents the KCCQ-PL and SF-12-PCS scores and the 5-level categorized variables (i.e., improved, declined, etc.), by gender. Women, in comparison to men, reported significantly lower mean PL scores (±SD) at baseline (28.7 ± 7.6 versus 30.8 ± 8.6; *P* = .01) and 12 months (30.3 ± 7.5 versus 34.2 ± 10.4; *P* < .01). Similarly, women reported significantly lower mean PCS scores (±SD) than men at baseline (45.4 ± 24.2 versus 52.1 ± 26.8; *P* = .01) and 12 months (51.0 ± 24.4 versus 61.5 ± 27.9; *P* < .01). There were no significant gender differences in the 5-level outcome variables for disease-specific and generic health outcomes at 12 months. Across the 12-month period, maintenance or improvement of functioning, as measured with either the PCS or PL, occurred in approximately 1/3 of the sample.

### 5.3. Social Support and Adverse Functional Outcomes

Tables 5 and 6 assess if social support predicts decline in functional outcomes after adjusting for age and sex. Overall, none of the social support domains was significantly associated with a decline in the SF-12-PCS; however, increasing levels of informational support, social and overall support were weakly and significantly associated with less decline in KCCQ-PL changes. Several sex-specific associations were identified. Men were less likely to experience disease-related functional decline with higher levels of affection support (OR .75, 95% CI .59, .96). When using the generic PCS functional outcome score as the dependent variable, levels of emotional/informational support (OR .70; 95% CI: .52, .93), affectionate support (OR .76; 95% CI: .59, .98), and positive social interaction (OR .78; 95% CI: .61, 1.00) in men were significantly associated with functional decline. There was an effect modification of gender on social support; males with high social support scores were less likely to report a functional adverse outcome, when using the PCS score as the basis for functional decline.

Sensitivity analysis, in which participants lost to followup were grouped with the functional declining group, resulted in similar findings to the aforementioned logistic regression models. Male participants were less likely to experience functional decline with more support; this was not the case for women. 

## 6. Discussion

The purpose of this study was to describe the independent and combined effects of gender and social support on one-year functional status in older persons with HF. Women consistently reported lower levels of functioning, but over the course of the year, following an exacerbation of their illness, both women and men experienced similar levels of functional maintenance or decline. The effect of social support on maintenance of function was limited to men, where men who perceived high levels of support experienced better outcomes.

The personal, social, and clinical characteristics of participants in this cohort study were similar to those of both older persons in general and to those with HF. Women, in comparison to men, were more likely to be single and on their own, with less income. A portrait of Seniors in Canada prepared by Health Canada in 2002 reported that nearly 75% of Canadian senior men compared to 41% of Canadian senior women were married, and 46% of senior women were widowed compared to 13% of senior men. Our study grouped single and divorced individuals with those who were widowed, unlike Health Canada that compared only widowed to those who were married. This difference in grouping is likely the reason for the comparatively higher percentage of participants in our nonmarried group and is of particular relevance to this study as we were exploring the availability of support regardless of reason. Also, as expected, men were more likely to have lower left ventricular ejection fraction. Generally men, regardless of age, are more likely than women to develop systolic dysfunction and typically have lower LVEF, more severe disease, and shorter survival times [25, 26] and, consequently, may require intense and shorter duration of support to optimize their functioning within the context of their progressive disease. However, since survival times are lengthier for women, they are living longer with HF than men and, therefore, may require more long-term support. 

### 6.1. Gender and Functional Status

Similar to other studies, our findings show that, compared to male participants, female participants reported significantly lower physical functioning, as measured by both disease-specific and generic measures and all participants reported low levels of functioning with only about one third of the sample maintaining or improving their functional level over the course of the year. In a cross-sectional, correlation study, Heo and colleagues [27] used the Duke Activity Status Index, a self-report tool, to assess the functional status of 122 HF patients. Out of a possible score ranging from 0 to 58, women reported lower functional status scores than men; mean scores (±SD) were 10.2 (±10.3) and 14.5 (±12.7), respectively (*P* = .04) [19]. Riedinger et al. [28] also demonstrated comparable results in a cross-sectional study of 1382 age and LVEF-matched HF patients [28]. When controlling for NYHA class, age, and LVEF, women had lower mean scores (SD) than men in measures of functional status including basic activities of daily living (ADLs) (*P* < .01), intermediate ADLs (*P* < .01), and perceived general health (*P* < .01). 

Qualitative research in the field has given valuable insight into patients' experiences with HF and its subsequent effects on their functional well-being [29, 30]. Bosworth et al. [29] identified 5 themes from a cross-sectional qualitative study of focus groups of male patients with HF. Symptoms, role loss, affective responses, coping, and social support were all areas patients identified as being negatively affected by their HF and consequently decreased their QOL. Similar themes were identified by Heo and colleagues [27] in an interview-based qualitative study of men and women living with HF. Participants identified personal and material supports from their significant others as having an important impact on their quality of life. Findings from our study suggest that both men and women experience considerable physical burden living with HF and that women report more limitations than men. 

### 6.2. Gender and Maintenance of Functional Well-Being

Despite the gender differences in functional levels, no differences existed in functional maintenance or decline over one year. These results are consistent with other studies. After adjusting for disease severity, although women rated QOL worse than men in a number of domains, Riedinger et al. [28] found no significant differences between genders with respect to QOL changes. Possible explanations for our findings include (1) women started with a lower functional score, perhaps less severe disease (i.e., higher LV function) with greater opportunity for improvement and less capacity for decline, whereas men started with higher scores, more severe disease and less capacity for improvement and more capacity for decline; (2) individuals who completed the 12-month data collection period were more likely to be functioning at a higher level to begin with and, as reflected in the reported 12-month scores, were more likely to maintain or improve functioning regardless of gender; (3) the disease progression of HF is difficult to influence, and so functional decline inevitably occurs. As reflected in the physical limitation scores of the KCCQ, more participants experienced disease-related functional decline versus overall functional decline in the physical component score of the SF-12. Unfortunately, the long-term prognosis for HF is poor, with 5-year survival rates for men and women being <40%; thus, functional decline, especially which relates directly to the disease and disease impact, is expected. 

### 6.3. Social Support and Functional Outcomes

Levels of social support at baseline had little impact on 12-month disease-specific functional outcomes; however, social support influenced 12-month generic functional outcomes. Men were significantly less likely to report a decline in general health or physical function, to drop out of the study due to increased illness, or to die within one year of an acute HF exacerbation when they perceived high levels of emotional/informational, affectionate, and positive social interactional support. No significant moderating effect of gender on social support and adverse outcomes were seen in women in this cohort. This is somewhat contradictory to findings reported in the literature. Similar to our study, previous literature supports the finding that increased social support has positive associations with health outcomes, however, the specifics of who benefits, and how they benefit differs between studies. Bennett et al. [2] found that the likelihood of a HF-related admission decreased by 10% for each unit increase in tangible support (*P* = .05) for both male and female participants of all ages [2]. In another study of older persons (≥65 years) with HF, absence of emotional support significantly increased the odds of cardiovascular events, defined as death or hospitalization due to cardiovascular disease, within one year of HF-related admission (OR 3.2; 95% CI: 1.4, 7.8) [5]. These associations were only found in women. Social support seems to exert an influence on selected functional outcomes and/or cardiovascular events, but the direction and strength of this influence is not clear, nor is it consistent between genders. Our study contributes novel findings about the influence of support on functionally related outcomes and the nature of the interactions between gender and support on these outcomes. Further investigation is needed to be able to identify the type and amount of support needed to assist both men and women in managing their HF. 

## 7. Strengths and Limitations

A particular strength of this study is the detailed data captured at baseline and followup on patients with HF, a population that is usually difficult to recruit and engage in study participation. As well, we employed standardized questionnaires, allowing for comparisons across reported studies. Self-report measures are valid measures of person's perception of their illness and are related to clinical outcomes [1]. Furthermore, where possible, we ensured that participants were able to complete the self-report questionnaires. However, we do acknowledge that questionnaire completion may have been compromised by the effect of age and/or heart failure on cognitive and other abilities. Another strength of our study is that our outcome of interest was based on clinically important functional changes. Results are therefore more clinically meaningful and relevant to practice. A 52% completion rate limits the result validity; 20% of the baseline participants did not complete one-year followup due to death, or illness which emphasizes the fragility of this population. We addressed patient attrition to some extent in the sensitivity analysis. Patient attrition likely diluted relationships found between outcomes and support; however, trends found for both PL and PCS-based outcomes were similar to that which we saw when the lost-to-followup group was excluded from the analysis. This would suggest that the relationships we found in a relatively stable HF sample could be an underestimate of the pattern in a more compromised sample. 

## 8. Conclusions

This study reports on the gender differences in social support and its corresponding relationship to both general and disease-specific adverse functional outcomes in the HF population. The results indicate that older women report less available social support and worse physical functioning both in relation to their general health and HF. In addition, the relationship between social support and adverse functional outcomes is seemingly moderated by gender in this cohort, with men less likely to experience a decline in their health outcomes with more perceived social support. This was not the case for women. These results also show that although women report less social support than men, social support may have less of a direct influence on health outcomes and physical function and that other supportive resources such as self-care capacity and availability of formal health care supports may have a stronger impact on physical function for women. Our findings support the need for gender-sensitive care for older HF patients and further research into the complex interactions between gender, supportive resources, and functional maintenance within the context of a chronic disabling condition such as HF.

## Figures and Tables

**Table 1:** Comparison of baseline characteristics of participants who completed 12-month questionnaires to those who were lost to followup or died.

	*n* = 224	*n* = 211	*P*
	Completers	Noncompleters	
Age			
Mean (SD)	77.5 (6.6)	78.7 (7.3)	.087
	*n* (%)	*n* (%)	
Gender			
Female	81 (36.2)	83 (39.3)	.495
Male	143 (63.3)	128 (60.7)	
Marital status			
Single/widowed/divorced	89 (39.7)	91 (43.1)	.472
Married/common-law	135 (60.3)	120 (56.9)	
Annual combined income			.067
≤$40,000	131 (64,5)	124 (73.4)	
>$40,000	72 (35.5)	45 (26.6)	
LV ejection fraction			.399
≤40%	96 (51.6)	84 (47.2)	
>40%	90 (48.4)	94 (52.8)	
Comorbid conditions			.193
0–2 comorbidities	113 (57.1)	98 (50.5)	
>2 comorbidities	85 (42.9)	96 (49.5)	

Social support subscale scores	Mean (SD)	Mean (SD)	

Emotional/information	74.1 (26.7)	70.2 (27.7)	.130
Tangible	78.2 (26.9)	75.5 (31.7)	.334
Affectionate	81.7 (28.1)	79.4 (30.2)	.417
Positive social interaction	75.0 (30.1)	67.1 (33.5)	.012
Overall	76.0 (24.7)	71.7 (27.1)	.082
Functional scores			
KCCQ PL Score	52.4 (25.3)	46.6 (26.4)	.026
SF-12-PCS	30.3 (8.3)	29.7 (8.3)	.455

**Table:** (a)

	Women	Men	*P*
	*n* = 164	*n* = 271	
Age			
(mean, SD)	78.1 (7.0)	77.8 (7.0)	.109
	*n* (%)	*n* (%)	
Marital status			
Single/widowed/divorced	108 (65.9)	72 (26.6)	<.001
Married/common-law	56 (34.1)	199 (73.4)	
Highest educational level			
Completed high school	127 (77.4)	210 (77.5)	.907
Completed postsecondary	33 (20.1)	53 (19.6)	
Missing	4 (2.4)	8 (3.0)	
Current living arrangements			
Living alone	75 (45.7)	53 (19.6)	<.001
Living with others	88 (53.7)	218 (80.4)	
Missing	1 (.6)	—	
Geographical distance from centre			
≤50 km	151 (92.1)	239 (88.2)	.198
>50 km	13 (7.9)	32 (11.8)	
Annual combined income			<.001
≤$40,000	111 (67.7)	144 (53.1)	
>$40,000	28 (17.1)	89 (38.8)	
Missing	25 (15.2)	38 (14.0)	
Use of other resources to manage HF^a^			.168
No	117 (71.3)	176 (64.9)	
Yes	47 (28.7)	95 (35.1)	

^a^ Use of resources to manage HF includes pamphlets, books, and/or the Internet.  a: resources include books, pamphlets, and the Internet.

**Table:** (b)

	Women	Men	*P*
	*n* = 164	*n* = 271	
Major criteria			
Paroxysmal nocturnal dyspnea	37 (22.8)	71 (26.6)	.385
Orthopnea	62 (38.0)	110 (41.4)	.496
Elevated jugular venous pressure	69 (42.9)	125 (47.3)	.367
Pulmonary rales	129 (79.6)	208 (72.9)	.673
Third heart sound	10 (6.1)	22 (8.3)	.402
Cardiomegaly	76 (46.6)	139 (52.3)	.258
Minor criteria			
Pulmonary edema on chest radiograph	76 (47.2)	107 (40.4)	.167
Peripheral edema	88 (54.3)	166 (62.2)	.109
Night cough	37 (23.1)	57 (21.4)	.683
Dyspnea on exertion	124 (77.0)	213 (80.4)	.408
Hepatomegaly	3 (1.9)	9 (3.4)	.359
Pleural effusion	60 (36.8)	104 (39.1)	.636
Heart rate > 120	18 (11.3)	34 (12.8)	.646
Wgt loss > 4.5 kg in 5 days in response to diuretics	3 (1.9)	10 (3.8)	
Framingham Criteria met^a^			.219
No	19 (11.6)	22 (8.1)	
Yes	143 (87.2)	248 (91.5)	
Missing	2 (1.2)	1 (.4)	
LV ejection fraction			.006
≤40%	54 (32.9)	126 (46.5)	
>40%	81 (49.4)	103 (38.0)	
Missing	29 (17.7)	42 (15.5)	
Comorbid conditions^b^			.068
0–2 comorbidities	72 (43.9)	139 (51.3)	
>2 comorbidities	78 (47.6)	103 (38.0)	
Missing	14 (8.5)	29 (10.7)	

^a^Diagnosis of CHF requires the simultaneous presence of at least 2 major criteria or 1 major in conjunction with 2 minor criteria which is outlined by the Framingham Criteria for congestive heart failure.

^b^The functional comorbidity index is an 18-item list of diagnoses associated with functional impairment.  Median number of comorbidities for both females and males was 2.0.

**Table 3:** Gender differences in baseline social support scores.

	Baseline		
	*N*	Mean (SD)	*P*
Emotional/information			.108
Women	163	69.5 (27.4)	
Men	265	73.9 (27.0)	
Tangible			.002
Women	162	71.3 (31.0)	
Men	266	80.3 (27.7)	
Affectionate support			.844
Women	161	80.9 (27.0)	
Men	265	80.4 (30.4)	
Positive social interaction			.057
Women	154	67.3 (31.7)	
Men	264	73.4 (32.0)	
Additional			.144
Women	158	67.6 (31.5)	
Men	263	72.3 (32.9)	
Overall			.068
Women	163	71.0 (24.6)	
Men	269	75.7 (26.6)	

**Table 4:** Baseline and 12-month functional scores and functional outcomes by gender.

	Women		Men		*P*
	*n*	Mean (SD)	*n*	Mean (SD)	
SF-12-PCS					
Baseline	160	28.7 (7.6)	263	30.8 (8.6)	.012
12 months	81	30.3 (7.5)	143	34.2 (10.4)	.003
KCCQ-PL					
Baseline	149	45.4 (24.2)	254	52.1 (26.8)	.012
12 months	80	51.0 (24.4)	137	61.5 (27.9)	.006

Functional outcomes	*n*	*n* (%)	*n*	*n* (%)	

SF-12-PCS change	164		271		.078
Maintained/improved		54 (32.9)		99 (36.5)	
Declined		13 (7.9)		20 (7.4)	
Died		12 (7.3)		42 (15.5)	
Too ill to participate		12 (7.3)		18 (6.6)	
LTFU/Missing		73 (44.5)		92 (33.9)	
KCQ-PL change	164		271		.121
Maintained/improved		46 (28.0)		72 (26.6)	
Declined		26 (15.9)		47 (17.3)	
Died		12 (7.3)		42 (15.5)	
Too ill to participate		12 (7.3)		16 (5.9)	
LTFU/Missing		68 (41.5)		94 (34.7)	

LTFU: loss to followup.

**Table 5:** Influence of social support on decline in SF-12-PCS.

	Missing data excluded		Missing data included	
	*n*	OR (95% CI)	*n*	OR (95% CI)
Emotional/ informational	217		428	
Overall		.99 (.98,1.02)		.99 (.99,1.00)
Female specific		1.12 (.79,1.59)		.97 (.76,1.24)
Male specific		**.70 (.52,.93)**		**.79 (.64,.96)**
Sex interaction^†^		**1.58 (1.01,2.49)**		1.24 (.90,1.70)
Tangible	219		428	
Overall		1.00 (.99,1.01)		1.00 (.99,1.00)
Female specific		1.22 (.86,1.72)		1.06 (.86,1.32)
Male specific		.80 (.59,1.06)		.86 (.71,1.04)
Sex interaction^†^		1.52 (.97,2.40)		1.24 (.93,1.65)
Affectionate	219		426	
Overall		1.00 (.99,1.01)		1.00 (.99,1.00)
Female specific		1.37 (.91,2.05)		1.18 (.93,1.50)
Male specific		**.76 (.59,0.98)**		**.82 (.69,.99)**
Sex interaction^†^		**1.78 (1.10,2.87)**		**1.43 (1.06,1.94)**
Positive social interaction	216		418	
All		1.00 (1.00,1.01)		.99 (.99,1.01)
Female		1.20 (.86,1.69)		.99 (.80,1.22)
Male		**.78 (.61,1.00)**		**.82 (.69,.97)**
Interaction		**1.53 (1.01,2.32)**		1.20 (.91,1.58)
Overall	212		432	
Overall		1.00 (.99,1.01)		1.00 (.98,1.01)
Female specific		1.28 (.84,1.95)		1.04 (.80,1.36)
Male specific		.76 (.57,1.02)		**.81 (.66,1.00)**
Sex interaction^†^		**1.68 (1.01,2.80)**		1.28 (.92,1.79)

*OR (95% CI): odds ratios with 95% confidence intervals estimated by multiple logistic regression adjusting for age and sex.  Each social support domain is modelled separately.  The ORs estimate the multiplicative increase in the odds of a MCID decline in PCS per 20-point increase in the social support score.  An OR < 1 indicates a protective effect of social support.  ORs statistically significant at *P* < .05 are in bold font.

^†^The sex interaction is the female-specific OR divided by the male-specific OR. An interaction OR > 1 indicates a greater protective effect of social support for males than females.

**Table 6:** Influence of social support on decline in KCCQ PL score.

	Missing data excluded		Missing data included	
	*n*	OR (95% CI)	*n*	OR (95% CI)
Emotional/ informational	269		428	
Overall		1.00 (.99,1.01)		**.99 (.98,1.00)**
Female specific		1.08 (.78,1.50)		.91 (.71,1.16)
Male specific		.83 (.66,1.05)		**.78 (.65,.98)**
Sex interaction^†^		1.28 (.86,1.90)		1.14 (.83,1.57)
Tangible	269		428	
Overall		1.00 (.99,1.01)		.99 (.99,1.00)
Female specific		0.99 (.75,1.31)		.95 (.77,1.18)
Male specific		0.95 (.73,1.23)		.82 (.67,1.00)
Sex interaction^†^		1.03 (.71,1.51)		1.16 (.87,1.56)
Affectionate	265		426	
Overall		0.99 (.99,1.00)		1.00 (.99,1.01)
Female specific		1.00 (.73,1.37)		1.08 (.85,1.37)
Male specific		**.75 (.59, .96)**		**.80 (.66, .97)**
Sex interaction^†^		1.31 (.88,1.95)		1.34 (.99,1.82)
Positive social interaction	260		418	
Overall		.99 (.99,1.00)		**.99 (.99,1.00)**
Female specific		.99 (.75,1.29)		.98 (.79,1.21)
Male specific		.82 (.73,1.10)		.79 (.66,.94)
Sex interaction^†^		1.20 (.85,1.70)		1.24 (.94,1.64)
Overall	271		432	
Overall		1.00 (.99,1.01)		**.99 (.98,.99)**
Female specific		.97 (.69,1.37)		.96 (.74,1.25)
Male specific		.87 (.67,1.12)		**.74 (.59,.92)**
Sex interaction^†^		1.11 (.73,1.71)		1.30 (.92,1.84)

^∗^OR (95% CI): odds ratios with 95% confidence intervals estimated by multiple logistic regression adjusting for age and sex.  Each social support domain is modelled separately.  The ORs estimate the multiplicative increase in the odds of a MCID decline in PL per 20-point increase in the social support score.  An OR < 1 indicates a protective effect of social support.  ORs statistically significant at *P* < .05 are in bold font.

^†^The sex interaction is the female specific OR divided by the male-specific OR. An interaction OR > 1 indicates a greater protective effect of social support for males than females.
